# Tuberculosis vaccines: beyond bacille Calmette–Guérin

**DOI:** 10.1098/rstb.2011.0097

**Published:** 2011-10-12

**Authors:** Helen McShane

**Affiliations:** Reader in Vaccinology and Wellcome Senior Clinical Research Fellow, The Jenner Institute, University of Oxford, Old Road Campus Research Building, Oxford OX3 7DQ, UK

**Keywords:** tuberculosis, vaccine, bacille Calmette–Guérin, clinical trials, efficacy, immune correlates

## Abstract

Tuberculosis (TB) disease caused by *Mycobacterium tuberculosis* (*M. tb*) remains one of the leading infectious causes of death and disease throughout the world. The only licensed vaccine, *Mycobacterium bovis* bacille Calmette–Guérin (BCG) confers highly variable protection against pulmonary disease. An effective vaccination regimen would be the most efficient way to control the epidemic. However, BCG does confer consistent and reliable protection against disseminated disease in childhood, and most TB vaccine strategies being developed incorporate BCG to retain this protection. Cellular immunity is necessary for protection against TB and all the new vaccines in development are focused on inducing a strong and durable cellular immune response. There are two main strategies being pursued in TB vaccine development. The first is to replace BCG with an improved whole organism mycobacterial priming vaccine, which is either a recombinant BCG or an attenuated strain of *M. tb*. The second is to develop a subunit boosting vaccine, which is designed to be administered after BCG vaccination, and to enhance the protective efficacy of BCG. This article reviews the leading candidate vaccines in development and considers the current challenges in the field with regard to efficacy testing.

## 1. Introduction

Tuberculosis (TB) is one of the leading global causes of death and disability from a single infectious agent, *Mycobacterium tuberculosis* (*M. tb*), with an estimated 9.4 million new cases and 1.7 million deaths in 2008 [1]. The Stop TB Partnership goals include reducing the global burden of TB (prevalence and mortality) by 50 per cent by 2015 compared with 1990 levels and eliminating TB as a public health problem by 2050 [2]. Prophylactic immunization is a key strategy in reducing the incidence of TB. *Mycobacterium bovis* bacillus Calmette–Guérin (BCG), the only licensed TB vaccine, is a live attenuated strain of *M. bovis* which was passaged by Calmette and Guérin almost one hundred years ago. BCG was first administered orally in 1921, and since then many clinical trials in different parts of the world have evaluated the efficacy of BCG in preventing TB disease. These trials demonstrate that BCG confers consistent protection against TB meningitis and disseminated TB in children, and leprosy in areas of the world where that disease is endemic [3–6]. However, BCG affords highly variable protection against pulmonary disease, which accounts for the major burden of global TB mortality and morbidity throughout the world [7]. Furthermore, revaccinating with BCG during adolescence in a population vaccinated with BCG at birth does not improve protective efficacy as shown in a large, randomized controlled trial (RCT) in Brazil [8]. BCG is currently administered in mass immunization campaigns to neonates in high-risk populations as part of the World Health Organization (WHO) Expanded Programme on Immunization (EPI). A more effective vaccine is a major global health priority.

In order to design an improved vaccine against TB, an understanding of the nature of protective immunity is required. An intact and robust cellular immune response is an essential prerequisite for protective immunity against mycobacterial disease, and all the new TB vaccines currently in development are directed towards inducing high levels of cellular immunity. Human leucocyte antigen (HLA) class II-restricted CD4^+^ T cells, together with the Th-1 cytokines interferon gamma (IFNγ) and tumour necrosis factor alpha (TNFα) are necessary for protective immunity [9–14]. Interleukin 2 (IL-2) is known to be important for central memory T cell responses [15]. HLA class I-restricted CD8^+^ T cells are probably also required for optimal protective immunity [16,17]. More recently, evidence is emerging for a protective role for CD4^+^ T cells that secrete IL17, Th-17 cells [18]. However, it is becoming increasingly clear that there may be a difference between aspects of immunity known to be necessary for protection, and an immune response that correlates with protection. In preclinical studies, IFNγ appears to be necessary but not sufficient for protection, and the magnitude of the response correlates with the degree of protection in some but not all studies [19–22]. Recent results from a large cohort of BCG-vaccinated South African infants have shown that the frequency of multi-functional T cells making IFNγ, TNFα and IL-2, 10 weeks post-vaccination was not associated with protection in this population [23]. However, any immunological correlate may be vaccine and disease-stage specific. Given the diversity of vaccine candidates being developed, and the diversity of clinical disease states, it is unlikely that a single, simple immune correlate exists across all these different populations.

Given the protection BCG does confer against disseminated disease in childhood, most new vaccine strategies being developed incorporate BCG, either by genetically engineering BCG to be more immunogenic, or by developing a subunit booster vaccine which is designed to be given after BCG vaccination. There are concerns regarding the safety of the existing BCG vaccine in HIV-infected infants, and some of the recombinant BCGs currently in development are designed to be safer in this population [24]. Both strategies can be combined, and a booster vaccine for an improved BCG could potentially be administered. The focus of this review is on new prophylactic TB vaccines which have progressed into clinical evaluation. The lead candidates that have progressed to clinical testing are summarized in table 1.

**Table 1.** TB vaccine candidates that have undergone or are in clinical development.

vaccine type	vaccine name	stage	key reference(s)
BCG replacements	rBCG30	phase I	Hoft *et al*. [25]
	VPM1002	phase I	Grode *et al*. [26]
	Aeras 422	phase I	Sun *et al*. [27]
	*Mycobacterium vaccae*	phase III efficacy	von Reyn *et al*. [28]
BCG boosters	M72	phase II safety	Von Eschen *et al*. [29]
	Hybrid I	phase II safety	van Dissel *et al*. [30]
	HyVAC IV	phase I	Dietrich *et al*. [31]
	Aeras 402	phase II safety and efficacy	Abel *et al*. [32]
	MVA85A	phase II safety and efficacy	McShane *et al*. [33], Scriba *et al*. [34]

## 2. Replacements for bacille Calmette–Guérin

### (a) Recombinant bacille Calmette–Guérin strains

There have been two recombinant strains of BCG that have been evaluated in clinical trials. The first rBCG30 was developed at the University of California, Los Angeles [35]. This vaccine candidate, engineered to over-express the 30 kDa major secreted antigen from *M. tb* was more protective than the wild-type strain in the guinea pig model. A phase I clinical trial with this vaccine demonstrated that this candidate was safe and immunogenic in humans [25]. This candidate is not currently in clinical development. A second approach to improving BCG is to generate a BCG strain that targets specific immune-processing pathways. A recombinant BCG strain, constructed to secrete listeriolysin, has been shown to be more protective than the wild-type strain in the murine model [26]. The proposed mechanism for the enhancement in efficacy with this vaccine candidate is by way of increased acidification of the phagosome, leading to antigenic escape to the cytoplasm and enhanced cross-priming of an HLA class I-restricted CD8^+^ T cell response. This vaccine has now been evaluated in a phase I clinical trial in Germany and is currently being evaluated in a phase IIa trial in South Africa (clinicaltrials.gov trials identifiers NCT00749034; NCT01113281). A combination of the two approaches described above is being pursued by the TB vaccine foundation Aeras, who have developed a recombinant strain of BCG expressing several antigens from *M. tb* together with perfringolysin [27]. This recombinant BCG is soon to enter into a phase I clinical trial in the US (http://www.aeras.org/portfolio/clinical-trials.php?id=17).

### (b) Attenuated strains of *Mycobacterium tuberculosis*

A second approach to improving BCG as a mycobacterial priming vaccine is to develop an attenuated strain of *M. tb*. Two groups are currently evaluating the safety and protective efficacy of attenuated strains of *M. tb* in preclinical models [36,37]. One of these vaccine candidates can confer levels of efficacy comparable with or superior to BCG in guinea pigs and non-human primates [36]. There are some safety concerns with the evaluation of attenuated strains of *M. tb* in clinical trials and two recent WHO workshops have addressed how this might best be achieved [38,39]. With detailed preclinical safety studies it is likely that at least one of these candidates will advance to early stage clinical testing in the next few years.

### (c) Mycobacterium vaccae

An inactivated whole cell strain of *Mycobacterium vaccae* (*M. vaccae*) was developed initially as a therapeutic TB vaccine candidate [40]. Variable results have been obtained in different geographical locations. There was no difference between treatment and placebo groups in a double blind RCT in South Africa [41]. *Mycobacterium vaccae* has since been evaluated as a prophylactic vaccine. One RCT of five doses of *M. vaccae* in BCG-vaccinated, HIV-infected patients in Tanzania demonstrated significant protection against the secondary endpoint of definite TB, although not against the primary endpoint of disseminated (bacteraemic) disease or against the other secondary endpoint, probable TB [28].

## 3. Bacille Calmette–Guérin booster vaccines

The main alternative strategy to replacing BCG is to leave BCG in its current form, administered in early infancy, and develop a booster vaccine, to be administered at a later point in time. Such a booster vaccine might either be administered in infancy, soon after BCG vaccination, or might be administered in adolescence, when the effects of BCG are starting to wane. Development of a subunit booster vaccine requires selection of both antigen(s) for inclusion in the vaccine and also identification of a suitable antigen delivery system. There are two main approaches to the development of a booster vaccine currently being pursued in the field. The first is to use a protein vaccine, in which case an adjuvant needs to be co-administered in order to induce high levels of cellular immunity. The alternative approach is to develop a recombinant viral vector, as some viruses themselves are an effective method of inducing strong cellular immunity.

### (a) Protein–adjuvant vaccines

#### (i) M72/MTB72F

One such recombinant protein is MTB72F (now remade as M72), a 72 kDa polyprotein of the *M. tb*32 and *M. tb*39 antigens, which is being developed by GlaxoSmithKline Biologicals (GSK). M72 is being delivered with the GSK adjuvants AS01 and AS02, which are a mixture of either a liposomal formulation (AS01) or a proprietary oil-in-water emulsion (AS02) with the immunostimulants monophosphoryl lipid A and *Quillaja saponaria* fraction 21. Preclinical efficacy studies with this protein–adjuvant combination have demonstrated efficacy comparable with BCG in mice and guinea pigs [42]. A phase I study with MTB72F and AS02A, administered as a three dose regimen in purified protein derivative (PPD)-negative healthy volunteers demonstrated a moderately reactogenic profile, with nine of 12 subjects (75%) enrolled experiencing a Grade 3 adverse event [29]. This trial demonstrated the induction of antigen-specific CD4^+^ T cells measured both by short-term enzyme-linked immunosorbent spot (ELISPOT) assay (where peripheral blood mononuclear cells (PBMC) are restimulated *in vitro* for one day prior to transferring to an ELISPOT plate) and flow cytometry. The median peak response measured on short-term ELISPOT assay, at day 56 (day of second vaccination), was approximately 100 spot-forming cells per million PBMC. IgG antibody responses were also demonstrated and also peaked at day 56. A further phase I study with this vaccine candidate demonstrated a similar safety profile and comparable CD4^+^ T cell responses, with no detectable CD8^+^ T cell responses [43]. This vaccine is currently being evaluated in phase IIa studies in South Africa (clinicaltrials.gov identifiers: NCT00600782; NCT00950612).

#### (ii) Hybrid I/HyVAC IV

Hybrid I is a fusion protein of two secreted antigens, the early secreted antigenic target 6 (ESAT 6) and antigen 85B, being developed by Statens Serum Institut, Copenhagen. In preclinical models, this fusion protein has been administered with the mucosal adjuvant LTK63, and leads to an improvement in BCG-induced protection in mice but not guinea pigs [44,45]. A phase I clinical trial with this fusion protein, administered with a novel adjuvant composed of an anti-microbial peptide and an immunostimulatory oligodeoxynucleotide, IC31 (Intercell, Vienna), demonstrated an acceptable safety profile and the induction of antigen-specific CD4^+^ T cells, as measured by short-term ELISPOT assay and enzyme-linked immunosorbent assay (ELISA) [30]. The peak short-term ELISPOT response in the high-dose group was approximately 600 spot-forming cells per million PBMC. However, the inclusion of ESAT6 in a subunit vaccine has the potential to confound the new generation of diagnostic tests which are increasingly in routine clinical use for the diagnosis of latent *M. tb* infection [46]. In this phase I study with Hybrid I, two of the 12 subjects (17%) in the high-dose group developed a positive Quantiferon gold response, owing to the ESAT6 antigen component of the fusion protein. In one of these subjects, this response remained positive at 131 weeks post-vaccination [30]. The replacement of ESAT6 with another immunogenic antigen from *M. tb*, TB10.4, in the next-generation vaccine, HyVAC IV, circumvents this issue [31]. Hybrid I is currently being evaluated in a phase I trial in Ethiopia (clinicaltrials.gov trial identifier NCT01049282) and HyVAC IV is currently being evaluated in phase I trials in Sweden and South Africa (http://www.aeras.org/portfolio/clinical-trials.php?id=19).

### (b) Recombinant viral vectors

#### (i) Aeras 402/Ad35-85B-TB10.4

This vaccine candidate is a recombinant, replication-deficient adenovirus, serotype 35, expressing a fusion protein created from the sequences of the antigens Ag85A, Ag85B and TB10.4 from *M. tb*. While recombinant adenoviral vectors are a potent way to induce cellular immunity, particularly an HLA class I-restricted CD8^+^ T cell response, pre-existing immunity induced by natural exposure to circulating adenoviral strains limits the utility of some strains, e.g. AdHu5 [47]. Pre-existing immunity to AdHu35 is known to be lower [48]. This vaccine is immunogenic and protective in mice and non-human primates [49,50]. A phase I clinical trial in BCG-vaccinated South African adults has demonstrated this vaccine to be safe and to induce high levels of predominantly monofunctional CD8^+^ T cells, with lower levels of polyfunctional CD4^+^ T cells [32]. This vaccine is currently being evaluated in a phase II safety and efficacy clinical trial in HIV-infected adults, in South Africa (clinicaltrials.gov identifier NCT01017536). A phase I/II safety and efficacy trial in BCG-vaccinated infants is also now underway (clinicaltrials.gov identifier NCT01198366).

#### (ii) Modified vaccinia virus Ankara 85A

This vaccine is a recombinant strain of modified vaccinia virus Ankara (MVA) expressing antigen 85A from *M. tb*. MVA is an attenuated strain of vaccinia virus, and was used at the end of the smallpox eradication campaign in Southern Germany [51]. It has an excellent safety profile and does not replicate in human tissue. Antigen 85A is part of the immunodominant antigen 85 complex, a component of which is part of many of the subunit vaccines in development. In preclinical studies, MVA85A can improve BCG-induced protection in mice, guinea pigs, non-human primates and cattle [22,52–54]. Cattle are relevant, not only as a preclinical animal model but also as a target species in their own right, as bovine TB is a significant problem throughout the world [55]. All four preclinical animal models allow the opportunity to identify potential immunological correlates of protection which can then be evaluated in clinical trials. In an *M. bovis* challenge experiment in cattle, BCG boosted with either MVA85A or AdHu5 expressing antigen 85A resulted in significantly more lesion free animals than BCG alone [22]. Cultured ELISPOT responses, presumed to measure central memory T cell responses [56], evaluated on the day of challenge were higher in the protected animals than in the diseased animals. Furthermore, levels of IFNγ and IL-17 measured by reverse transcriptase real-time–polymerase chain reaction (RT-PCR) on the day of challenge also correlated with protection in these animals.

**Figure 1.**
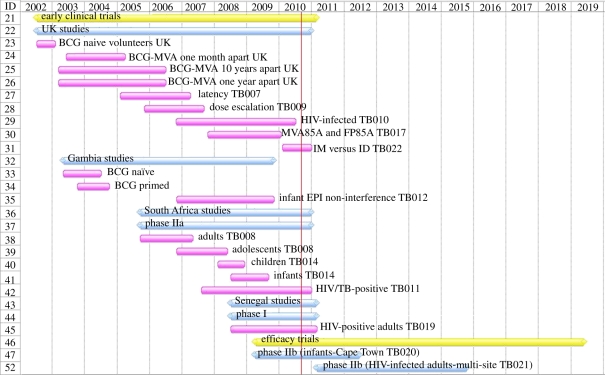
Gantt chart summarizing clinical trials with MVA85A since 2002.

MVA85A was first evaluated in a phase I study in 2002. Since then, 12 clinical trials have been completed and four more are underway. Figure 1 is a Gantt chart that summarizes these clinical trials. This vaccine is currently the most clinically advanced new TB vaccine, and is discussed in greater detail below in order to illustrate the development path for new TB vaccines.

When MVA85A first entered into clinical evaluation in 2002, there was considerable concern within the field of TB vaccines regarding the potential induction of immunopathology, or a so-called Koch reaction, in people who were latently infected with *M. tb*. This concern arose from Robert Koch's original experiments with the ‘cure’ he developed, which was essentially culture filtrate from *M. tb*. This ‘cure’ induced severe reactions in some of the TB patients whom he injected, with some fatalities [57]. A similar effect of immunopathology has since been demonstrated in preclinical animal models with a high burden of disease, but does not seem to occur in animals with low bacillary burdens corresponding with latent *M. tb* infection [58]. For this reason, the trials with MVA85A were commenced in subjects believed to be as ‘mycobacterially naive’ as possible, i.e. tuberculin skin test negative, BCG-naive healthy subjects [33]. Once safety and immunogenicity had been demonstrated in this group, trials in BCG-vaccinated subjects were conducted. The results of these early studies demonstrated that MVA85A was safe and highly immunogenic, and that the antigen-specific CD4^+^ T cell response induced was significantly higher in BCG-vaccinated subjects, than in BCG-naive subjects [33]. Further studies demonstrated that the interval between BCG priming and boosting with MVA85A did not appear to be critical, and comparable boosting was achieved whether BCG was administered many years or one month prior to boosting [59]. Results from these early studies also demonstrated the induction of Class I-restricted CD8^+^ T cells, and that the CD4^+^ T cell responses induced were highly polyfunctional [60,61]. Subsequent studies in *M. tb* latently infected subjects demonstrated safety and equivalent immunogenicity in this group to that seen in BCG-vaccinated subjects [62].

Once sufficient safety data had been generated from the UK studies, phase I/IIa clinical trials with MVA85A commenced in TB endemic countries, first The Gambia, and then South Africa and most recently Senegal. Studies in Gambian adults demonstrated safety and immunogenicity, although interestingly the immunogenicity in BCG-naive and BCG-vaccinated subjects was comparable in this population [63]. Studies in South African adults confirmed these safety and immunogenicity results, and again immunogenicity in BCG-naive and BCG-vaccinated subjects was comparable [64]. It is likely that the contribution of BCG vaccination to cumulative mycobacterial immunity in adults living in tropical climates is significantly less than in more temperate climates, and that cumulative exposure to non-tuberculous mycobacteria and *M. tb* contributes significantly to the resulting total anti-mycobacterial immunity. Further results from the South African age de-escalation studies have demonstrated safety and the induction of a highly polyfunctional CD4^+^ T cell response after vaccination in adults, adolescents and children [34,64]. These vaccine-induced responses were sustained at levels significantly higher than baseline until the last follow-up visit in the trial. A more recent study has evaluated the effect of co-administration of the routine Expanded Programme on Immunization childhood vaccines with MVA85A in BCG-vaccinated Gambian infants, to determine any immunological interference with either the cellular immune response induced by MVA85A or the humoral immune response induced by the EPI schedule vaccines (clinicaltrials.gov Identifier NCT00480454).

HIV-infected adults are significantly more susceptible to TB disease than their HIV-uninfected counterparts, and thus represent an important target population for an improved TB vaccine [65]. Clinical trials with MVA85A are currently underway in HIV-infected adults in the UK, South Africa and Senegal (clinicaltrials.gov trial identifiers NCT00395720, NCT00480558 and NCT00731471). It is important to demonstrate safety in this population and in particular to evaluate any effect of vaccination on HIV RNA load and CD4 count, as well as evaluating vaccine-induced immunogenicity.

## 4. Efficacy testing

In every clinical trial conducted to date with MVA85A, this vaccine has demonstrated an excellent safety profile. The cellular immunity induced by MVA85A is known to be essential for protective immunity, even if the individual cytokine responses have not been shown to correlate with protection. Furthermore, in preclinical models, MVA85A can improve BCG-induced protection. However, the most important question is: ‘is MVA85A effective at preventing TB disease in people?’ This is a difficult question to address. In the absence of immunological correlates of protection that would allow us to predict with some certainty which vaccine candidates would be effective, and in the absence of a validated animal model which is known to predict efficacy in humans, we are left with clinical efficacy trials as the only way to answer this question for this and all the other vaccines in clinical development. Such clinical efficacy trials must be carried out in areas of the world with the highest incidence of disease, but even so require large numbers of subjects and long periods of follow-up. Such trials are hugely resource intensive and there are only a few clinical trial sites in the world where such trials are currently possible. Robust and detailed epidemiological data are required in order that the efficacy trials are powered accordingly. Considerable infrastructure is required, not only in terms of facilities but also in terms of clinical trial expertise, at these sites. There are three main target populations for an improved vaccine against TB: infants, adolescents and HIV-infected adults. In these latter two populations, such a vaccine may also need to work as a post-exposure vaccine in latently infected individuals. These three populations are mycobacterially and immunologically very different and require different expertise, and potentially a vaccine might be effective in one of these populations but not another.

A phase IIb efficacy trial evaluating MVA85A in BCG-vaccinated South African infants commenced enrolment in July 2009 (clinicaltrials.gov trail identifier NCT00953927). This is a double blind RCT, where 18–26-week-old infants are randomized to receive either MVA85A or placebo. The objectives of this trial are safety, in expanded numbers, immunogenicity in a subset of subjects, and efficacy, both against infection and against disease. A second phase IIb efficacy trial in HIV-infected adults is scheduled to commence in 2011 (clinicaltrials.gov trail identifier NCT01151189). This study will also evaluate safety, immunogenicity and efficacy against both disease and infection. Two phase I/II safety and efficacy trials with Aeras 402 are also underway in South Africa in HIV-infected adults (clinicaltrials.gov trails identifier NCT01017536) and infants (clinicaltrials.gov trails identifier NCT01198366).

Importantly, in both of these efficacy trials, samples are being stored from all subjects in order that these can be used for immune correlate studies at the end of the trial. These samples are a unique and valuable resource for the identification of potential immunological correlates of protection. Such studies are essential to facilitate the development of TB vaccine in the future. The power of such efficacy trials to attempt to identify immune correlates has been demonstrated by a large RCT of BCG vaccination in infants in South Africa [66]. A nested case-control study was embedded in the design of this clinical trial, and samples taken 10 weeks after BCG vaccination were stored for the identification of immune correlates. To date, levels of polyfunctional CD4^+^ T cells in these samples do not correlate with protection and further ongoing work is investigating many other aspects of cellular immunity using both immunological assays and gene expression studies [23]. One caveat here is that any immunological correlate may be vaccine- and disease-stage specific.

## 5. Summary

In the past 10 years, there has been substantial progress in the field of TB vaccine development. In 2010, there were 11 candidate vaccines being evaluated in clinical trials, compared with none 10 years previously. Despite concern 10 years ago, there have been no safety issues or immunopathology identified with any of the candidate vaccines currently being evaluated. Two of these candidates, MVA85A and Aeras 402, are currently being evaluated in efficacy trials. In the absence of immune correlates and predictive animal models, efficacy testing is currently the only way to evaluate whether any of the new generation of vaccines prevents TB disease in humans. Protective immunity against mycobacterial disease is a complex interaction between the innate immune response, the Th1, Th2, Th17 pathways and regulatory T cells (Treg). In the absence of an immune correlate, there is a need for better models with which to evaluate the efficacy of new TB vaccines. Several preclinical animal models have utility and it is necessary to show some efficacy in at least some of these models before progressing into clinical trials. However, all of these preclinical models fail to represent the human situation in important ways, (i) BCG confers consistent protection in these models (unlike in humans) and (ii) most of these models are relatively simple pre-exposure models where all animals develop disease, and efficacy is measured as a reduction in colony forming unit (CFU) counts. Until we have an effective vaccine in humans, we will not be able to determine how representative these preclinical models are in predicting efficacy in humans.

The challenges for the next 10 years are clear. It is unlikely that any simple immunological correlate of protection will be identified. There is a limited capacity to conduct these large efficacy trials, both in terms of available clinical trial sites and in terms of resources available to fund such trials. Therefore, it is essential that we develop tools to select which vaccines should progress. This lack of reliable, relevant models with which to select which vaccines should go forward into these large-scale trials is the single most significant challenge within the field of TB vaccine development. As more vaccine candidates progress from preclinical models to early stage clinical testing, there will be an increasing need for the development of *in vivo* and *in vitro* models with which these candidates can be evaluated. Once established, such models would initially need validating in efficacy trials, by demonstration that efficacy in such a model correlates with efficacy in human efficacy trials. Such a model would then be of great utility in enabling a rational selection of vaccines for entry into these large efficacy trials.
